# Impact of combined sodium chloride and saturated long-chain fatty acid challenge on the differentiation of T helper cells in neuroinflammation

**DOI:** 10.1186/s12974-017-0954-y

**Published:** 2017-09-12

**Authors:** Anna Hammer, Anne Schliep, Stefanie Jörg, Aiden Haghikia, Ralf Gold, Markus Kleinewietfeld, Dominik N. Müller, Ralf A. Linker

**Affiliations:** 1 Department of Neurology, University Hospital Erlangen, Friedrich-Alexander-University Erlangen-Nürnberg, Schwabachanlage 6, 91054 Erlangen, Germany; 2 Department of Neurology, Ruhr-University Bochum, 44801 Bochum, Germany; 3 VIB Laboratory of Translational Immunomodulation, VIB Center for Inflammation Research (IRC), Hasselt University, BIOMED, 3590 Diepenbeek, Belgium; 4 Experimental and Clinical Research Center, a joint cooperation of Max-Delbrück Center for Molecular Medicine and Charité-Universitätsmedizin Berlin, 13125 Berlin, Germany; 5 Max-Delbrück Center for Molecular Medicine in the Helmholtz Association, 13125 Berlin, Germany; 6 Berlin Institute of Health (BIH), Berlin, Germany

**Keywords:** T helper cell, Diet, Salt, Saturated fatty acids, Multiple sclerosis, EAE, Inflammation

## Abstract

**Background:**

There has been a marked increase in the incidence of autoimmune diseases like multiple sclerosis (MS) in the last decades which is most likely driven by a change in environmental factors. Here, growing evidence suggests that ingredients of a Western diet like high intake of sodium chloride (NaCl) or saturated fatty acids may impact systemic immune responses, thus increasing disease susceptibility. Recently, we have shown that high dietary salt or long-chain fatty acid (LCFA) intake indeed aggravates T helper (Th) cell responses and neuroinflammation.

**Methods:**

Naïve CD4^+^ T cells were treated with an excess of 40 mM NaCl and/or 250 μM lauric acid (LA) in vitro to analyze effects on Th cell differentiation, cytokine secretion, and gene expression. We employed ex vivo analyses of the model disease murine experimental autoimmune encephalomyelitis (EAE) to investigate whether salt and LCFA may affect disease severity and T cell activation in vivo.

**Results:**

LCFA, like LA, together with NaCl enhance the differentiation of Th1 and Th17 cells as well as pro-inflammatory cytokine and gene expression in vitro. In cell culture, we observed an additive effect of LA and hypertonic extracellular NaCl (NaCl + LA) in Th17 differentiation assays as well as on IL-17, GM-CSF, and IL-2 gene expression. In contrast, NaCl + LA reduced Th2 frequencies. We employed EAE as a model of Th1/Th17 cell-mediated autoimmunity and show that the combination of a NaCl- and LA-rich diet aggravated the disease course and increased T cell infiltration into the central nervous system (CNS) to the same extent as dietary NaCl.

**Conclusions:**

Our findings demonstrate a partially additive effect of NaCl and LA on Th cell polarization in vitro and on Th cell responses in autoimmune neuroinflammation. These data may help to better understand the pathophysiology of autoimmune diseases such as MS.

## Background

The increasing incidence of autoimmune diseases with a high prevalence in Western countries [1] and the rapid evolution of multiple sclerosis (MS) in former low prevalence countries like Japan [2] indicate a role of environmental factors that do play a role beyond predisposing genetic risk factors. Here, most impressively, dietary habits like the so-called Western diet, high in both fat and salt, may play a role (as reviewed in [3, 4]).

High dietary salt (sodium chloride (NaCl)) intake is a major culprit in cardiovascular disease [5], cancer [6], chronic inflammation [7], and also autoimmune diseases [8, 9]. Mimicking in vivo situations in the tissue after a high-salt diet by raising sodium concentrations in vitro has been shown to affect adaptive immune cells and promote the differentiation of murine and human T helper (Th)17 cells with a pathogenic phenotype [10, 11]. Here, the serum- and glucocorticoid-regulated kinase-1 (SGK1) is one of the key mediators of salt-augmented differentiation of Th17 cells. Further studies on the effects of NaCl-induced hypertonicity on the activation and function of myeloid dendritic cells (DC) provide evidence that, in autoimmune neuroinflammation, high-salt effects on T cells are rather directly exerted than primarily mediated via DC [12]. In experimental autoimmune encephalomyelitis (EAE), a Th1/Th17 model disease mimicking many features of MS [13], a high-salt diet augmented disease onset and severity and correlated with enhanced breakdown of the blood-brain barrier (BBB) as well as more severe brain pathology [10, 11]. Exacerbated disease was accompanied by increased induction of Th17 cells and elevated numbers of central nervous system (CNS)-infiltrating pathogenic Th17 cells. In MS patients, a recently published study demonstrated an enhanced disease activity, relapse risk, and increased magnetic resonance imaging (MRI) activity in subjects with an increased dietary sodium intake [9].

Similar to a high-salt diet, an increased intake of long-chain fatty acids (LCFA), like lauric acid (LA), exacerbated autoimmunity in the CNS [14]. This was due to an increased infiltration of Th1 and Th17 cells in the spinal cord. In vitro, the differentiation of murine and human CD4^+^ T cells into Th1 and Th17 cells was significantly increased by the addition of LCFA. In murine EAE, diets rich in LCFA were shown to modulate the microbiome, exposing CD4^+^ T cells to increased LCFA concentrations in the small intestine, thus inducing pro-inflammatory T cell responses [14].

Of note, the consumption of “Westernized food,” including high-salt, high-fat, high-protein, and high-sugar intake, has already been associated with an increased prevalence of various diseases [15, 16]. However, there is still little understanding on the mechanisms linking environmental factors to disease mechanisms, genetic predisposition, and the immune system. Here, we investigated whether a combination of increased NaCl and LA may have additional effects on CD4^+^ T cell populations in vitro and in vivo, thus further underlining the relevance of a “Western diet” as a risk factor for autoimmune diseases.

## Methods

### Isolation of naïve T cells

Splenic T cells were isolated by magnetic-activated cell sorting using the “pan T cell isolation kit II” according to the manufacturer’s instructions (Miltenyi Biotech, Bergisch Gladbach, Germany). Cells were fluorescently stained with an antibody cocktail containing αCD4-FITC (RM4-5, eBioscience, San Diego, CA), αCD44-PE (IM7, BioLegend, San Diego, CA), αCD62L-APC (MEL-14, eBioscience), and αCD25-PECy5 (PC61.5, eBioscience) and were subsequently isolated by fluorescence-activated cell sorting on MoFlo (Beckman-Coulter, Brea, CA) in the FACS core unit in Erlangen, Germany.

### T cell culture and differentiation

Sorted naïve T cells (CD4^+^CD62L^+^CD44^low^CD25^−^) were stimulated by plate-bound anti-CD3 (2 μg/ml, 145-2C11, BD Pharmingen, San Diego, CA) and soluble anti-CD28 (2 μg/ml, 37.51, BD Pharmingen) and cultured for 4 days in the presence of interleukin (IL)-6 (40 ng/ml, R&D Systems, Minneapolis, MN) and IL-23 (20 ng/ml, BioLegend) for Th17, IL-12p70 (20 ng/ml, R&D Systems) and anti-IL-4 (10 μg/ml, BioLegend) for Th1, or IL-4 (50 ng/ml, Miltenyi Biotech) and anti-interferon gamma (IFNγ; 10 μg/ml, BioLegend) for Th2 differentiation. To determine the influence of LA and NaCl on T cell differentiation, cells were cultured with and without 250 μM LA and/or an excess of 40 mM NaCl. T cell frequencies were analyzed by flow cytometry (FACSCantoII, BD Biosiences, San Jose, CA).

### Animal experiments

Mice strains were backcrossed on C57BL/6J background for at least 10 generations. All mice were housed at the Präklinisches Experimentelles Tierzentrum (PETZ), the animal care facility of the University Erlangen-Nürnberg, under a 12-h day-night cycle and standardized environmental conditions receiving normal chow (SNIFF E15431-34EF R/M; 0.4% NaCl, 4.2% crude fat) and tap water ad libitum. All experiments were in accordance with the German laws for animal protection and were approved by local ethic committees (Erlangen AZ 54-2532.1-56/12 and 55.2 DMS 2532-2-27).

For experiments under the high-salt diet, mice received chow containing 4% NaCl (based on SNIFF E15431-34EF) and tap water containing 1% NaCl. Mice were adapted to high-salt chow for 4 weeks prior to induction of active myelin oligodendrocyte glycoprotein (MOG)_35–55_ EAE. For experiments under high-fat diet, mice received a chow containing 30.9% crude fat, rich in LA (13.47%; based on SNIFF E15431-34EF). Mice were adapted to high-fat chow 4 weeks before EAE induction. For experiments under high-salt and high-fat diet, mice received a chow containing 30% crude fat and 4% NaCl (based on SNIFF E15431-34EF) and tap water containing 1% NaCl. Mice were adapted to high-salt + high-fat chow 4 weeks before EAE induction.

For EAE induction, 10–12-week-old mice were anesthetized and subcutaneously injected with 200 μg MOG_35–55_ and 200 μg Complete Freund’s Adjuvant (CFA), containing 4 mg/ml *Mycobacterium tuberculosis* (H37RA, BD Biosciences). Pertussis toxin (200 ng/mouse, List Biological Laboratories, Campbell, CA) was applied intraperitoneally on days 0 and 2 post immunization (p.i.). Daily clinical evaluation was performed via a 5-point scale.

### Isolation of splenic cells

Spleens were removed on day 10 p.i. and disrupted with a 5-ml glass homogenizer. The tissue was then filtered through a 100 μm cell strainer followed by an erythrocyte lysis. After washing with cold PBS, cells were processed by intracellular cytokine staining and ex vivo flow cytometry analysis.

### Isolation of CNS-infiltrating cells

The spinal cord was removed on day 21 p.i. and disrupted with a 5-ml glass homogenizer. Tissue was strained through a 100 μm cell strainer followed by a three-step density gradient (30, 45, and 70% isotonic Percoll™, GE Healthcare, Uppsala, Sweden). After centrifugation (20 min at 800*g* without brake), cells were harvested from the interphases, washed with cold PBS, and re-suspended in medium for intracellular cytokine staining and ex vivo flow cytometry analysis.

### In vitro MOG restimulation assay

Splenocytes from EAE mice were obtained on day 10 p.i. and seeded at a density of 6 × 10^6^ cells/ml. MOG_35–55_ (20 μg/ml) and concanavalin A (1.25 μg/ml) were added for stimulation, and supernatants were harvested 48 h later and analyzed for cytokine production.

### Flow cytometry

Ex vivo-obtained splenic cells, CNS-infiltrating cells, and in vitro-differentiated T cells were analyzed by staining for extra- and intracellular markers. Dead cells were excluded by a fixable viability dye eFluor®780 (0.2 μl/test, eBioscience). Nonspecific Fc-mediated interactions were blocked by the addition of 0.5 μl αCD16/32 (93, eBioscience). For surface staining, the cells were treated with the respective fluorochrome-conjugated antibodies: αCD4-FITC (RM4-5, eBioscience) and αCD25-APC (PC61.5, eBioscience). For intracellular cytokine staining, cells were stimulated for 4 h with ionomycin (1 μM, Sigma-Aldrich, St. Louis, MO) and PMA (50 ng/ml, Sigma-Aldrich) in the presence of monensin (2 μM, eBioscience), fixed with 1% paraformaldehyde and made permeable by saponin or Fix/Perm buffer (eBioscience) treatment. Intracellular cytokines were stained with the respective fluorochrome-conjugated antibodies: αFoxP3-PE (FJK-16s, eBioscience), αIFNγ-APC (XMG1.2, eBioscience), αIL-17A-PE (eBio17B7, eBioscience), IL-4-PE (11B11, BioLegend), and GATA3-PerCP/eFluor710 (TWAJ, eBioscience). Probes were measured with a flow cytometer (FACSCantoII, BD Biosiences).

### Cytokine detection

Cytokine concentrations in cell culture supernatants were analyzed by enzyme-linked immunosorbent assays for the secretion of IL-17A and IFNγ (DuoSet ELISA Kits, R&D Systems, Minneapolis, MN) according to the manufacturer’s instructions.

### Real-time PCR

Total RNA was isolated using the RNeasy kit (Qiagen, Venlo, Netherlands) and reversely transcribed into cDNA using the QuantiTect® Reverse Transcription Kit (Qiagen). PCR reactions were performed with a qTower real-time PCR System (Analytik Jena, Jena, Germany). Relative quantification was performed by the ΔΔCT method, normalizing the target gene expression on *actb*/β-Actin as a housekeeping gene. The following TaqMan® real-time PCR assays from Thermo Fisher Scientific were used: actb (β-Actin) Mm00607939_s1, ahr Mm00478932_m1, csf2 Mm01290062_m1, il2 Mm00434256_m1, il6 Mm99999064_m1, il17 Mm00439618_m1, il23a Mm01160011_g1, rorc Mm01261019_g1, sgk1 Mm00441380_m1, tbx21 Mm00450960_m1, and tnf Mm00443258_m1.

### Statistical analysis

Statistical analysis was performed using GraphPad Prism (GraphPad Software Inc., La Jolla, CA). All in vitro and ex vivo data were analyzed by one-way ANOVA followed by Tukey’s post-test, unpaired *t* test, or Wilcoxon rank sum test after checking for normal distribution (unless indicated otherwise in the legends). EAE data were analyzed by Kruskal-Wallis test. Data are presented as mean ± SEM; *p < 0.05, **p < 0.01, or ***p < 0.001 were considered to be statistically significant.

## Results

### NaCl and LA display additive effects on the differentiation of Th17 cells

**Fig. 1:**
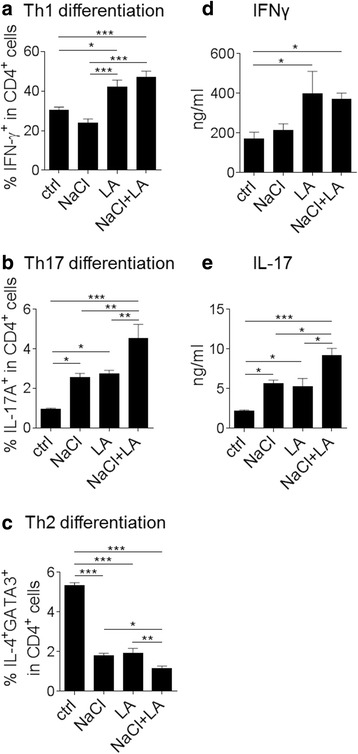
Combined NaCl + LA challenge promotes polarization of naïve T cells towards Th17 cells in vitro. **a** Addition of either 40 mM NaCl, 250 μM LA, or both to CD4^+^ T cell differentiation culture under Th1-polarizing conditions (data pooled from five independent experiments, *p < 0.05, ***p < 0.001). **b** IFNγ production of Th1 cells treated with either 40 mM NaCl, 250 μM LA, or both (data pooled from four to five independent experiments). **c** Addition of either 40 mM NaCl, 250 μM LA, or both to CD4^+^ T cell differentiation culture under Th17-polarizing conditions (data pooled from three independent experiments, *p < 0.05, **p < 0.01, ***p < 0.001). **d** IL-17 production of Th17 cells treated with either 40 mM NaCl, 250 μM LA, or both (three to four independent experiments are shown). **e** Addition of either 40 mM NaCl, 250 μM LA, or both to CD4^+^ T cell differentiation culture under Th2-polarizing conditions (data pooled from three independent experiments, *p < 0.05, **p < 0.01, ***p < 0.001)

To investigate a potential additive effect of a combination of NaCl and LA on the differentiation of naïve T cells under Th1, Th17, or Th2 cell-polarizing conditions in vitro, we added either 40 mM NaCl, 250 μM LA, or both to murine CD4^+^ T cell cultures (Fig. 1a–e). In accordance with published data, both NaCl and LA fostered the differentiation of Th17 cells (Fig. 1c) by around 50% as compared to control conditions whereas only LA increased the frequency of Th1 cells (Fig. 1a). Moreover, NaCl and LA diminished Th2 differentiation significantly (Fig. 1e). NaCl together with LA (NaCl + LA) further enhanced the differentiation of Th17 cells but reduced Th2 frequencies by about one third versus NaCl or LA alone (Fig. 1c, e). No additional effect of NaCl + LA was observed for Th1 cell differentiation compared to LA treatment alone (Fig. 1a). NaCl and LA also increased the production of the signature cytokines IFNγ (Fig. 1b) and IL-17 (Fig. 1d) in the supernatants of Th1 or Th17 differentiating cultures, respectively. In accordance with Th17 cell frequencies, an additional effect of NaCl + LA was observed for the production of IL-17 in culture supernatants (Fig. 1d).

### NaCl and LA challenge alters the expression profile of Th1 and Th17 cells

**Fig. 2:**
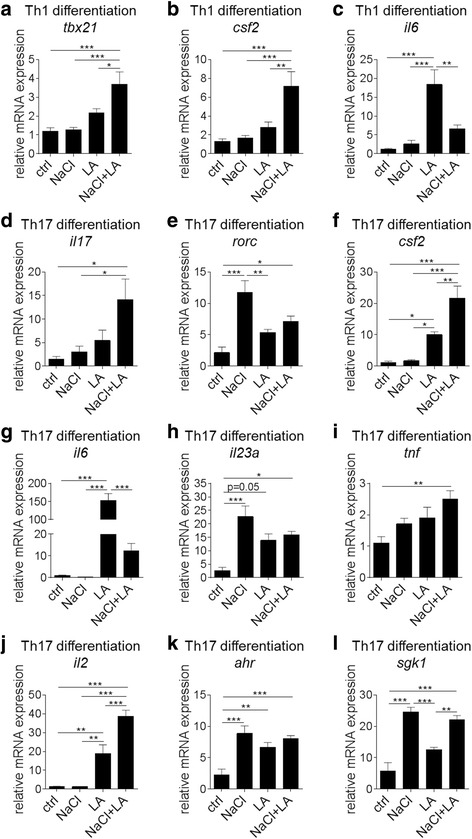
NaCl and LA exert distinct effects on Th1/Th17 cell gene expression profiles in vitro. **a**–**c** Gene expression analysis of *tbx21* (T-bet) (**a**), *csf2* (GM-CSF) (**b**), and *il6* (**c**) in Th1 differentiation assays (data pooled from four preparations, *p < 0.05, **p < 0.01, ***p < 0.001). **d**–**l** Gene expression analysis of *il17* (**d**), *rorc* (RORγ) (**e**), *csf2* (GM-CSF) (**f**), *il6* (**g**), *il23a* (**h**), *tnf* (**i**), *il2* (**j**), *ahr* (**k**), and *sgk1* (**l**) in Th17 differentiation assays (two out of four independent preparations are shown, *p < 0.05, **p < 0.01, ***p < 0.001)

Expression analyses of Th1 or Th17 cell gene signatures revealed increased mRNA expression of *tbx21* (T-bet) and *csf2* (granulocyte macrophage colony-stimulating factor (GM-CSF)) in NaCl + LA-treated Th1 cells compared to controls, NaCl, or LA treatment (Fig. 2a, b). In contrast, the *il6* expression was most significantly enhanced by LA alone (Fig. 2c).

In differentiated Th17 cells, NaCl as well as LA and NaCl + LA treatment increased the expression of a number of pro-inflammatory genes (Fig. 2d–l). An additional effect of NaCl + LA versus NaCl or LA alone was observed for *il17* (Fig. 2d), *csf2* (Fig. 2f), and *il2* (Fig. 2j) gene expression supporting the increased generation of Th17 cells under high NaCl + LA conditions in vitro. Aryl hydrocarbon receptor (*ahr*) expression in Th17 cells was similarly increased by NaCl, LA, and NaCl + LA (Fig. 2k), whereas expression of *sgk1*, as a known key mediator of salt-augmented activation of Th17 cells, was mainly induced by NaCl or NaCl + LA treatment (Fig. 2l).

### NaCl + LA-rich diet impacts on Th1 and Th17 cell-mediated CNS autoimmunity in vivo

**Fig. 3:**
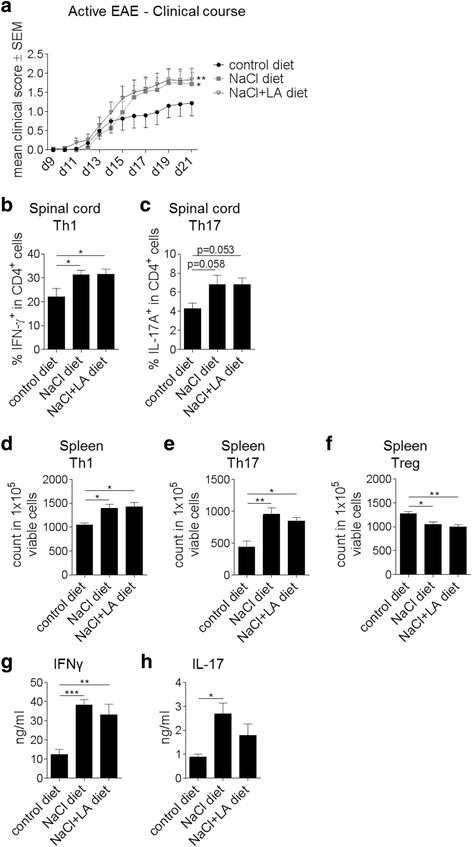
Combined high-fat and high-salt diet has no additional effect on EAE severity and immune cell activation. **a** Clinical course of MOG_35–55_ EAE. Mice were fed either a NaCl-rich or a NaCl + LA-rich diet for 4 weeks prior to immunization. Data are shown on a 5-point score scale (*n* = 15–20 mice per group, data pooled from four independent experiments, *p < 0.05). **b**, **c** Ex vivo flow cytometry analysis of Th1 (**b**) and Th17 (**c**) cell frequencies in the spinal cord under a NaCl-rich or NaCl + LA-rich diet versus the control diet on day 21 of MOG_35–55_ EAE (*n* = 9–13 mice per group, data pooled from three independent experiments, *p < 0.05). **d**–**f** Ex vivo flow cytometry analysis of Th1 (**d**), Th17 (**e**), and Treg (**f**) cell frequencies in the spleen under a NaCl-rich or NaCl + LA-rich diet versus the control diet on day 10 of MOG_35–55_ EAE (*n* = 4–5 mice per group, *p < 0.05, **p < 0.01). **g**, **h** Cytokine analyses in splenocyte culture after ex vivo recall with MOG_35–55_ (splenocytes harvested on day 10 p.i. of MOG_35–55_ EAE, *n* = 4–5 per group, *p < 0.05, **p < 0.01, ***p < 0.001)

The distinct in vitro effects of NaCl + LA on naïve CD4^+^ T cells prompted us to examine their effects in vivo using murine MOG_35–55_ EAE as a model of Th1/Th17 cell-mediated autoimmunity. C57BL/6J mice were fed standardized and otherwise completely matched diets rich in either NaCl or NaCl + LA and were compared to mice on a control diet after EAE induction. Mice on the NaCl + LA-rich diet did not display different body weights compared to those on the NaCl-rich or control diet (mean ± SEM on day 21 of EAE, 22.2 ± 0.65 g NaCl + LA diet versus 20.5 ± 0.85 g NaCl diet versus 22.0 ± 0.74 g control diet). Mice fed the NaCl- or NaCl + LA-rich diet displayed a more severe disease course compared to controls (Fig. 3a), although disease incidence and mortality were unaffected. Upon ex vivo phenotyping of spinal cord infiltrates by flow cytometry, the NaCl as well as NaCl + LA diet increased Th1 (Fig. 3b) and Th17 (Fig. 3c) cell frequencies in the CNS on day 21 p.i.. However, in contrast to our in vitro experiments, we observed no additional effect of a NaCl- and LA-rich diet on disease severity or CNS cell infiltration compared to a NaCl-rich diet (Fig. 3a–c). Analysis of the spleen showed enhanced Th1 (Fig. 3d) and Th17 (Fig. 3e) but decreased Treg (Fig. 3f) cell counts in mice fed a NaCl- or NaCl + LA-rich diet compared to the control group. Well in line with our previous data, a NaCl- and LA-rich diet had no additive effect on T cell counts (Fig. 3d–f). Ex vivo recall assays revealed an increase of the pro-inflammatory cytokines IFNγ and IL-17A in splenocytes derived from EAE mice on a NaCl-rich or NaCl + LA-rich diet compared to mice fed a control diet (Fig. 3g, h).

## Discussion

Recent epidemiological studies reveal that lifestyle factors such as smoking, obesity, and salt intake may constitute risk factors for MS [17, 18]. LCFA, like LA, or NaCl alone are already established environmental triggers of T cell differentiation that may act as risk factors for Th1 and/or Th17 cell-mediated autoimmune diseases like MS [11, 12, 14]. Yet, a so-called Western diet is typically rich in both, NaCl and LCFA. Thus, our present data offer an important extension to the already existing immunological and functional groundwork and epidemiological observations in MS patients and identify a combined challenge of saturated LCFA and NaCl together as additional dietary, non-infectious triggers involved in T cell differentiation.

Here, we show that NaCl + LA treatment exerts direct effects on Th1, Th2, and Th17 cells under polarizing conditions in vitro and, most importantly, enhances Th17 cell differentiation to a much greater extent than LA or NaCl alone. The concentration of sodium in the plasma is approximately 140 mM, similar to standard cell culture media. However, in the interstitium and lymphoid tissue, considerably higher sodium concentrations may be encountered. This setting is mimicked by adding an excess of 40 mM NaCl to cell culture media which is able to induce pathogenic Th17 cells [11, 19]. Thus, this dietary challenge may be a mechanism for decreasing immune activation in the blood while favoring a pro-inflammatory response in lymphoid tissues. With a regulatory role of Th2 responses in autoimmune neuroinflammation [20], a shift from Th2 towards Th1 cell polarization may add to the detrimental effects of a high-salt and/or high-fat diet alongside with opposing effects on Th17 and Treg cells. The lack of an additional effect of a combined LA and NaCl challenge on Th1 cells may be explained by several factors. All NaCl-induced effects on immune cells so far have been ascribed to Th17 cells [11], Treg cells [21], and macrophages including M2 polarization [22]. No comparable outcomes on the differentiation of Th1 cells were observed [11]. These studies support the concept that, in contrast to fatty acids, distinct immune cell subtypes respond differently to NaCl and that the salt-induced generation of a pro-inflammatory environment involves specific effects on immune cells rather than unspecific activation of all lymphocytes and antigen-presenting cells [12]. Therefore, in contrast to Th17 cell differentiation, NaCl- and LA-induced signaling cascades are not cooperative to further induce Th1 polarization.

Furthermore, the responsible receptors mediating effects of NaCl or LA remain to be identified. Given the plasticity of Th cells and their complex, divergent intercellular signaling pathways during differentiation, it is likely that several mechanisms act in concert. In addition to mitogen-activated protein (MAP) kinase family members, SGK1, which is critically involved in Th17 differentiation [10], was differentially expressed in LA- as well as NaCl-challenged T cells under Th17 cell-polarizing conditions.

In the setting of autoimmunity, a NaCl- and LA-rich diet contributed to a more severe course of EAE and enhanced Th1 and Th17 infiltration into the CNS to the same extent as a NaCl-rich diet alone. It is well conceivable that the pro-inflammatory microenvironment generated by dietary NaCl in immune compartments of the body cannot be further boosted by the addition of LCFA leading to similar disease severity in the animal model.

Although no long-term clinical trials currently exist, our data suggest that the cellular and molecular immunological basis underlying the impact of dietary components like saturated fats and sodium on inflammatory processes and autoimmunity deserves further investigation. While no definite associations between dietary restrictions and the modulation of autoimmune diseases have been firmly established yet, a large proportion of patients already consider special diets or dietary supplements as alternative therapeutic measures. In the first place, the efficacy of dietary interventions in autoimmune diseases may crucially depend on how well the disease pathology is controlled by established immunomodulatory and anti-inflammatory treatment options. Thus, further studies combining standard immunotherapy with dietary approaches in patients are highly warranted.

## Conclusions

LCFA, like LA, together with excessive NaCl enhance the differentiation of Th1 and Th17 cells but reduce Th2 frequencies. For Th2 and Th17 cells, an additive effect of LA and NaCl was observed in differentiation assays and on IL-17, GM-CSF, and IL-2 gene expression. In the EAE model, a combination of a NaCl- and LA-rich diet aggravated the disease course and increased T cell infiltration into the CNS to the same extent as dietary NaCl. In summary, different nutritional components may exert independent effects on distinct cells of the immune system but may also influence the functioning of these cells via cooperative mechanisms. These data further underline the relevance of a “Western diet” as a risk factor for MS. Our findings may help to better understand the pathophysiology of MS, thus ultimately enabling protocols for dietary intervention studies in neuroinflammation.

## Abbreviations

ahr: Aryl hydrocarbon receptor
BBB: Blood-brain barrier
CFA: Complete Freund’s Adjuvant
CNS: Central nervous system
EAE: Experimental autoimmune encephalomyelitis
GM-CSF: Granulocyte macrophage colony-stimulating factor
IFNγ: Interferon gamma
IL: Interleukin
LA: Lauric acid
LCFA: Long-chain fatty acids
MOG: Myelin oligodendrocyte glycoprotein
MRI: Magnetic resonance imaging
MS: Multiple sclerosis
NaCl: Sodium chloride
RORγ: RAR-related orphan receptor gamma
SGK1: Serum- and glucocorticoid-regulated kinase-1
T-bet: T-box-containing protein expressed in T cells
Th: T helper
Tnf: Tumor necrosis factor
